# A Pilot Randomised Trial Investigating the Effects of Including Efficacy Messaging on Tobacco Warning Labels

**DOI:** 10.1093/ntr/ntac229

**Published:** 2022-10-04

**Authors:** Lillian Brinken, Stuart G Ferguson, Marie-Jeanne Buscot, Benjamin Schüz, Olivia Maynard, Natalie Schüz

**Affiliations:** University of Tasmania, Hobart, Australia; University of Tasmania, Hobart, Australia; University of Tasmania, Hobart, Australia; University of Bremen, Bremen, Germany; University of Bristol, Bristol, UK; Deutsche Rentenversicherung Oldenburg-Bremen, Bremen, Germany

## Abstract

**Introduction:**

Smokers can respond defensively to health risk communication such as on-pack warning labels, potentially reducing their effectiveness. Theory suggests that risk perception together with self-efficacy reduces defensive responses and predicts target behaviors. Currently, tobacco warning labels globally predominantly target risk and do not explicitly consider efficacy.

**Aims:**

This study explores the effectiveness of combining Australian tobacco warning labels with efficacy content to increase quitting intentions.

**Methods:**

RCT in 83 smokers over 3 weeks. After a seven-day baseline phase (smoking from usual tobacco packaging), participants were randomized to one of two adhesive labels groups for the remaining 14 days: Standard health warning labels (HWLs) featuring enhanced efficacy messages (experimental group) or unmodified standard HWLs (control group). Participants attached these labels to their tobacco packaging and recorded their cognitions and smoking behavior once daily using Smartphones. Multilevel structural equation modeling was used to test theorized effects of the labels on self-efficacy, risk perception, and intentions to quit.

**Results:**

There was no effect of exposure to efficacy messages on either self-efficacy, risk perceptions, or intentions to quit. However, self-efficacy and risk perceptions were positively associated with quitting intentions at the within-person level.

**Conclusions:**

The predictive relationships between self-efficacy, risk perception, and intention to quit were supported, however, supplementing standard warning labels with efficacy messages had no effect on these cognitions. Whether this is due to conditioned avoidance of HWLS, characteristics of the messages, or limitations imposed by format are unclear.

**Implications:**

Self-efficacy and risk perception predict intentions to quit smoking. Adding efficacy content to tobacco health warnings may have the potential to bolster these cognitions but more research is required to determine the contexts in which this would be effective and who would be likely to benefit. The time course by which exposure to efficacy content might influence cessation self-efficacy and downstream quitting intentions also needs to be investigated.

## Introduction

Health warning labels (HWLs) first appeared on tobacco packaging in the United States in the 1960s.^1^ Despite industry opposition, legislation has been implemented across a growing number of countries mandating the diffusion of explicit and increasingly graphic HWLs. Guidelines for the implementation of tobacco HWLs have been established by the World Health Organisation’s Framework Convention on Tobacco Control,^2^ are required by the European Tobacco Products Directive,^3^ and have come to be considered a standard component of the tobacco control policy toolkit.

At their most basic, HWLs consist of a text-only warning on a section of an otherwise traditionally branded tobacco package. Such “text-only” warnings typically describe a particular threat to mortality or health caused by smoking (eg “Smoking cigarettes can cause lung cancer”). More stringent legislation—such as that enacted in Australia—requires these text warnings to be accompanied by images depicting the suffering and disease caused by tobacco.

The purpose of including warnings on tobacco packaging is firstly to prevent smoking uptake and secondly to catalyze smoking cessation; they are proposed to do this by increasing the readers’ perceptions of the health risks caused by smoking and inducing negative emotional arousal.^4,5^ Evidence generated through a range of methodological approaches supports the effectiveness of HWLs in achieving these aims^6^ and while it is difficult to quantify, there is some circumstantial evidence to suggest that the introduction of these warnings has contributed to decreased smoking prevalence in some countries.^7,8^ However, experimental evidence is less conclusive: for example, a more recent randomized controlled trial found that while quitting cognitions increased, no significant changes in health concerns or smoking behavior were observed among smokers exposed to HWLs compared to traditionally branded packaging.^9^

Despite the widespread adoption of HWLs in their current forms, theories of health behavior change suggest the effectiveness of health risk communication could be further increased by targeting smokers’ beliefs about their abilities to quit.^10^ As noted above, HWLs typically use the health risks associated with smoking to induce negative affect and increase perceptions of vulnerability, thus aiming to increase the likelihood of cessation by positioning quitting as a means of reducing these feelings of vulnerability and negative affect.^11^ However, theorists have long since noted the potential for threat to elicit defensive reactions rather than adaptive risk mitigation, as for example rationalizing or denying the existence of a health threat could also ameliorate such feelings of vulnerability without the need for behavior change.^12^ Here, the authors argue that caution needs to be taken when communicating threatening health information to prevent defensive responses such as avoidance, denial, or reactance which are associated with maladaptive health behaviors. In the case of tobacco HWLs, such maladaptive health behaviors could include rejection or minimization of the message, reduced interest in quitting, or even increasing smoking.

The Extended Parallel Process Model proposes that defensive processes leading to maladaptive behavior are likely when people are confronted with information about a health threat that they do not feel they can adequately protect against, resulting in attempts to reduce negative emotion rather than risk (so-called “fear control”).^13^ These responses may range from attentional biases such as the tendency to avoid looking at health warnings observed in eye-tracking studies,^14^ through to more elaborate processes such as downplaying personal susceptibility to death and disease^15^ or dismissing health threat communication as attempted manipulation.^16^ Such defensive responses towards threatening health risk communication are more likely to occur among populations particularly at risk of poor health outcomes and among those with low efficacy beliefs.^17^ Conversely, EPPM proposes that adaptive responses to health risk communication are more likely when belief in one’s own ability to mitigate threat and the benefit of doing so is high (a response referred to as “danger control”; Witte: 1992).^13^ Evidence supporting the predictions of the EPPM has been reported in other health-behavior contexts.^18,19^ This has led to calls for efficacy beliefs to be considered when designing a policy that targets health risk via the provision of threatening health information.^10,12^

Incorporating efficacy information into HWLs was recently explored in a mixed-methods study involving roll-your-own tobacco smokers in New Zealand. Participants were shown mockup tobacco pouches with efficacy content provided on the internal flap. The authors concluded that integrating efficacy content into the packaging design of tobacco pouches may lead to emotional reactions, beliefs, and behavioral intentions associated with quitting.^20^ While these findings are broadly consistent with the predictions of the EPPM, the nature of this study did not allow for the interpretation of any causal effects but was rather concerned with exploring smokers’ reactions to a single exposure in the context of tobacco packaging. Indeed, it has been shown that the effects of on-pack graphic information on health-related cognitions may wane with repeated exposure over time.^21^ Thus, the level of exposure that might be required to produce a meaningful shift in efficacy beliefs among smokers has not been established. Further, it has been suggested that smoking cessation (which involves extended abstinence in the face of environmental and physiological drivers) might be harder to influence than the enactment of infrequent behaviors which have been successfully targeted through single-exposures to health risk communication such as vaccination or screening.^22,23^ More controlled, experimental research is thus required to explore the possibility of improving the effectiveness of HWLs by including efficacy messages.

To determine whether exposure to efficacy content on tobacco HWLs is associated with danger control efforts (increases in quitting intentions), and whether this relationship is mediated by self-efficacy beliefs or perceptions of risk, we designed a two-arm RCT study with daily measurements using Ecological Momentary Assessment (EMA) methods. We specifically tested whether exposure to efficacy content on HWLs is associated with increases in quitting intentions, and, secondly, whether this relationship is mediated by self-efficacy beliefs or perceptions of risk.

## Methods

### Overview

This study used an intensive longitudinal, two-arm RCT design to study smoking-related cognitions over a three-week period. After a seven-day baseline phase, participants were randomized to use either standard HWL, or modified HWL that were enhanced with efficacy messages. Participants were given a supply of adhesive HWLs specific to the group to which they were allocated and then continued to monitor their smoking and smoking-related cognitions for an additional 2 weeks. In addition to the real-world monitoring, participants attended a total of three in-person laboratory visits. The study received ethical approval from the Human Research Ethics Committee (Tasmania) Network (Ethics Reference Number: H0015696) and written informed consent was obtained from all participants.

## Participants

The study was advertised via fliers posted in public places and on Facebook.^24^ Interested smokers completed an online expression-of-interest form and were subsequently screened over the phone by study staff to ensure that they met inclusion criteria. Those who had smoked for the previous 3 years, had no intention to quit in the next 3 months, and currently smoked a minimum of 10 cigarettes daily were invited to enroll. Pregnant women and those under the age of 18 years were excluded from participation for ethical reasons and shift workers were excluded as the survey protocol required a typical day–night sleep cycle. A sample of 83 smokers were recruited. The average participant was female (*n* = 44, 53% of the sample), 33 years old, Caucasian (*n* = 77, 93% of the sample), and reported smoking 16.7 (SD = 6.6) cigarettes per day (CPD) at baseline. Almost three-quarters (73.4%) reported smoking their first cigarette of the day within 30 min of waking. Forty-eight participants (58%) were randomized to the control (standard HWL) group; there were no differences in baseline characteristics between the two groups.

## Procedures

At their first lab visit, participants completed some baseline surveys about demographic information and smoking behaviors, and provided a sample of exhaled air to verify smoking status (recording greater than 7 ppm carbon monoxide breath sample as measured by piCO Simple Smokerlyzer^25^ was taken as evidence of being a smoker) and were then issued with Smartphones that had been stripped of their original functionality in favor of a custom EMA data collection application (HBART; https://www.utas.edu.au/health/research/groups/tasmanian-school-of-medicine/clinical/behavioural-and-situational-research-group-bsrg/hbart). Consistent with other EMA studies of smoking,^26–28^ participants were instructed to use these devices to log—by tapping a button on the screen—all cigarettes smoked during the study in real-time, respond to surveys issued by the device at random times (average of 4–5 per day) and complete scheduled surveys each morning and evening. The EMA device randomly selected 4–5 cigarettes logged per day for a full assessment. All EMA surveys could be completed in less than a minute.

Each day, participants were required to complete an evening report, rating their quitting intentions, risk perceptions, and self-efficacy beliefs. The items used to assess quitting intentions and self-efficacy beliefs were derived from surveys published in previous studies and measured participants’ agreement with a series of statements on scales of 1–10 and 1–9 respectively. Risk perceptions were measured on a scale of 1–7. Details of these questions can be found in the supplementary materials. During these end-of-day reports, participants were also asked to specify which of the Australian HWLs was currently on their tobacco packaging and were required to take a photograph of this HWL using the device.

**Figure 1.**
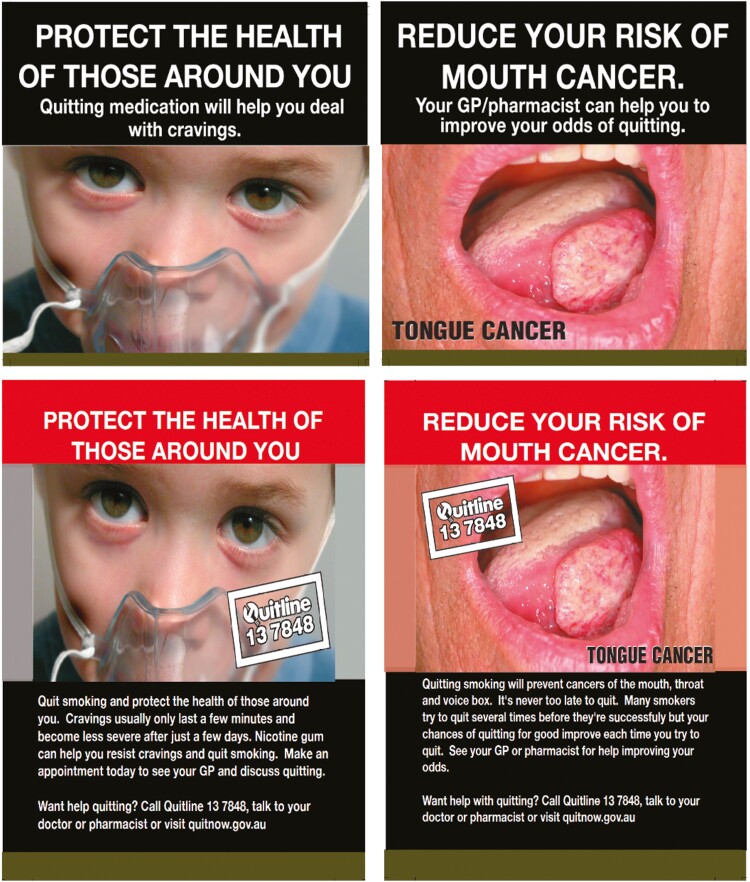
Examples of labels given to participants in the experimental group. Labels for the front of pack (top row) featured short slogans and subtitles which were expanded in line with the theme of the image on the back of pack (bottom row). “Don’t let others breathe your smoke” otherwise known as “the boy with the oxygen mask” used with permission from the European Union; “Smoking causes mouth cancer” used with permission from Dr Christopher Hughes on behalf of St Vincent’s Hospital, Sydney.

After a week of baseline monitoring, participants returned to the lab for a second study visit. At this visit, data were downloaded from devices and additional training on the monitoring procedures was provided to participants with poor compliance (at or below 80% completion of surveys issued) by study staff. Next, participants were randomized (un-blinded) to either the control or experimental groups and received adhesive labels for the front and back of their cigarette packs. Participants in the experimental group received adhesive HWLs that were modified from the current HWLs in Australia: Here, we used seven of the images in circulation in Australia (see Figure 1). To control for attention effects caused by using adhesive labels, participants in the control group received adhesive HWLs identical to those currently required by the Australian government to be displayed on tobacco packaging. The line of text along the top of the image on the front of the pack was modified to feature brief efficacy messages, aiming to increase participants’ confidence in their ability to quit and the effectiveness of quitting as a means of mitigating smoking health risks. The longer text paragraphs on the back of the packs expanded on the health risks and efficacy themes displayed on the front and provided contact details of the telephone support service mandated to appear on tobacco packaging in Australia. Participants were shown how to attach the HWLs to any tobacco packaging subsequently purchased and were given sufficient HWLs to last the duration of the experimental phase of the study.

At the end of 14-day experimental phase participants completed their third lab visit, returned their EMA devices and were debriefed. Participants were reimbursed for their time with vouchers which were issued at each study visit (AUD$90 in total). Compliance with EMA protocol was incentivized by offering an additional $20 voucher for participants who answered at least 80% of the random prompt surveys issued.

## Data Analyses and Analytic Plan

**Figure 2.**
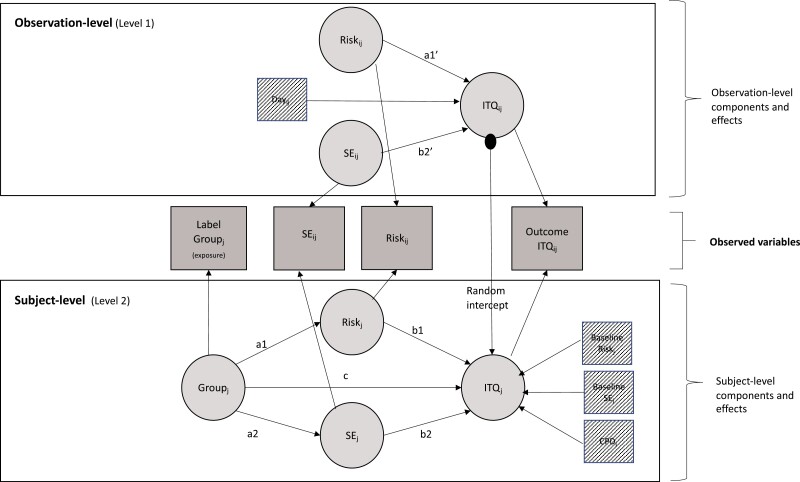
Model showing pathways and covariates. Covariates shown with striped fill. Abbreviations: SE = Self-efficacy; ITQ = Intention to quit, CPD = Cigarettes per day.

The objective of the study was to determine whether exposure to efficacy content on tobacco HWLs is associated with increases in quitting intentions, and whether this relationship is mediated by self-efficacy beliefs or perceptions of risk (Figure 2).

**Figure 3.**
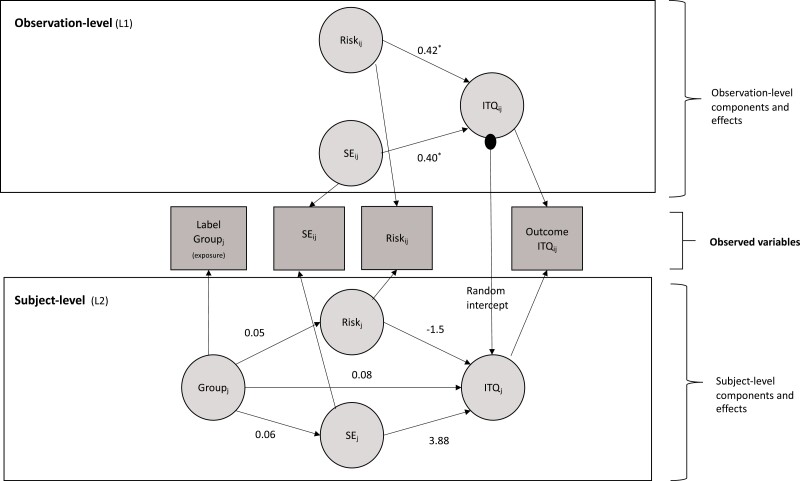
Multilevel Structural Equation model with parameter estimates. Abbreviations: SE = Self-efficacy; ITQ = Intention to quit. * = *p* < .05, ** = *p < .*01.

To investigate the effects of the intervention over time, it is crucial to disentangle within-person- from between-person effects, as in our study design, “treatment group” (ie exposure to modified HWL) was a person-level (level 2) variable, while both the potential mediators and the outcome of interest were measured repeatedly within-person over time (observation level [level 1] variables). Therefore, we used 2-1-1 multilevel mediation models (Figure 3) that distinguish between direct and mediated effects of a given exposure, while simultaneously allowing for the disaggregation of within-cluster (level-1) and between-cluster (level-2) variability in the outcome (latent decomposition).^29,30^

Daily measures of self-efficacy beliefs, risk-perception, and quitting intentions (level-1 variables) were mean-centered within-subject.^30^ Over the course of the study, the 83 study participants completed 978 such daily measures. The model examined the relationship between variables during the 14-day experimental phase (ie post-randomization); observations gathered during the pre-randomization period were used to generate participant-level baseline control variables of self-efficacy beliefs, risk-perception, that were used as participant-level covariates in the final model (see Figure 2 for details). The final model was also adjusted for baseline CPD (at level 2), and for the day since randomization (varying from 1 to 14) at level 1 (to test if there was any systematic way in which time affected key variables across the study period). All analyses were conducted in R with the *Lavaan* package^31^. The expectation-maximization algorithm was used to fit the model, and the estimates presented are bootstrapped parameters.

## Results

Overall compliance with daily monitoring post-randomization was high (89.7% over the 14 days; SD = 16.4%). Similarly, individual compliance with daily monitoring was also consistently high.

The results from the model are shown in Figure 3. Both the comparative fit index (CFI = 1.00) and root mean square error of approximation (RMSEA = 0.00; 90% CI *=* 0.00 to 0.06) were indicative of the model being a good fit for the data.

At the person level (level-2), the treatment group did not predict self-efficacy or risk perception, nor did it predict intention to quit directly, or indirectly through self-efficacy or risk perception (Figure 3). Participants with higher confidence in their ability to quit did not have higher intentions to quit than those with lower confidence in their ability to quit smoking. Further, those who perceive themselves to be at a higher risk of harm from smoking did not have higher quitting intentions than those who perceive their risk to be low.

However, there were positive direct effects of self-efficacy and risk perception on intention to quit at the observation level (level-1), with a one-unit increase in self-efficacy and risk perception respectively increasing intention to quit levels by 0.40 and 0.42; that is, on days when participants’ level of confidence in their ability was higher than their personal mean, they were more likely to also have higher quitting intentions. Similarly, on days when participants’ perception of smoking-related risk was higher than their usual estimation, they were more likely to have higher intentions to quit. There were significant negative effects of time both during the baseline and experimental phases which were similar in size (*γ*_01_ ranging between −0.02 and −0.04, *p =* < .05) suggesting a slight decrease in each scale both before and after randomization. Models including day-by-group interactions at level 2 indicated that the group did not significantly modify the effect of day on any of the variables.

## Discussion

This small pilot study set out to evaluate the effectiveness of using modified HWLs to increase self-efficacy beliefs as a way of influencing quitting intentions. We used EMA to examine relationships between constructs theorized to influence quitting (risk-perception and self-efficacy) and intentions to quit at both the within- and between-participant levels. Statistically speaking, we did not find any direct effect of exposure to warnings with efficacy content on intentions to quit smoking (research question 1), nor was there an effect of the intervention on self-efficacy beliefs or risk perceptions; variables predicted to mediate the relationship between threat communication and quitting intentions (research question 2). Further, at the between person-level, no associations between self-efficacy or risk perception and intention to quit were observed, indicating that people who report higher levels of these cognitions are not more likely to have higher quitting intentions than those who feel less efficacious or perceive less risk. Having said this, it is worth noting that the relationships between treatment group self-efficacy, risk perception, and intention to quit were consistent and in the predicted direction, though the parameter estimates were small and not statistically significant. Treatment group also predicted intention to quit indirectly through self-efficacy; again, however, the parameter estimates were nonsignificant.

We did observe the expected relationships between risk perception and self-efficacy as predictors of intention to quit at the within-person level. On days when participants reported having greater confidence in their ability to quit or felt themselves to be at a greater risk of harm (relative to their own personal means), they were more likely to also have higher intentions to quit. This observation was consistent with predictions made by the EPPM,^13^ but these relationships were not affected by the intervention. Together, these signals observed warrant exploration in a larger, adequately powered study.

Exposing smokers to efficacy content via tobacco packaging has been shown to increase behavioral intentions linked to quitting^20^ and studies examining the use of inserts with efficacy content have found an association between viewing messages and increases in self-efficacy and quitting intentions and attempts.^32^ However, these studies used observational methodologies that were not designed to identify experimental effects and looked at between- rather than within-person associations. The current study featured a control group, and an extended baseline phase, and used EMA to examine daily relationships via an intensive longitudinal design, allowing for changes over time in the relationships between cognitions to be captured. As such, this design was able to look at changes prospectively and attenuate some of the biases associated with measuring cognitions retrospectively^33^—but the modified warning labels used in the current study failed to produce an increase in efficacy beliefs, risk perception, or intentions to quit.

One possible explanation is that the specific messages we used were not able to bolster efficacy beliefs. When developing the efficacy messages used in this study, there was minimal literature to provide empirical or theoretical guidance around what might constitute an effective efficacy message. Messaging was further constrained by the space and format of existing Australian tobacco labeling and the need to draw a thematic link between efficacy content and the graphic images featured on labels. Development was informed by literature recommending the use of gain-framed messaging and interrogative questioning,^34,35^ however, the scope of the current study did not allow for these messages to be piloted prior to being rolled out in this RCT, which was itself a pilot study. The decision to modify existing HWLs for the experimental group and compare these to a control group with HWLs identical to those in circulation was made to maximize ecological validity and prevent extraneous design features influencing participant responses. However, because the HWLs resembled the government-mandated warnings so closely, it is possible that learned inattention or deliberate avoidance of health warning space affected the extent to which participants engaged with the efficacy content.^14,36^

The amount of time or the level of exposure to efficacy content required to shift cognitions was another factor which was difficult to predict. The three-week period of the study used here with two-weeks’ exposure was in line with other studies looking at the effects of tobacco packaging/warnings^28,37^ and intends to balance the burden of participation against the potential of uncovering the effects of repeated exposure over a time. However, to date, there has been scant evidence comparing the impact of interventions using different timeframes in the short to medium term and the EPPM does not specify the role of time in efficacy appraisals.^38^ It seems likely that boosting efficacy beliefs around behaviors such as smoking which are maintained by diverse personal and environmental factors would require a more extensive intervention than infrequent risk reduction measures such as cancer screenings or vaccinations. However, exactly how long such an intervention would need to remain to be seen and may be influenced by the smoking characteristics of the population, with factors such as readiness to quit previously observed to predict the effectiveness of efficacy and threat messaging in terms of quitting intentions.^39^ It is possible that those who are considering quitting or planning to do so may benefit more from efficacy content than smokers who intend to continue smoking^40^ (de Vries et al. 1998). In the current study, we wanted to explore the effects of efficacy content on smokers with no plans to quit in the immediate future and this was stipulated in the recruitment process.

Although the current study did not induce any significant changes to self-efficacy beliefs or risk perception, the study observed both theoretically and practically relevant relationships between self-efficacy, risk perception, and intention to quit on the within-person level: On days on which smokers felt more confident in their ability to quit, and on days when they perceived higher risk to their health compared to their usual levels, they also experienced higher intentions to quit. These findings provide support for the idea that these cognitions fluctuate over time and might be successfully manipulated in a different context and supports the theoretical rationale for investigating these variables further as potential targets to catalyze cessation; it is possible, for example, that for efficacy messages might be most impactful when they overlap with a smoker’s natural fluctuations (as opposed to by gradually changing these cognitions directly over time).

These findings also underscore the necessity of research methods which can capture shifts in the relationships between health cognitions over time and allow for the separation of between- and within-persons effects. More research is required in this field to identify what constitutes a successful efficacy message, how efficacy content can best be deployed in a public health context, and which populations would be most likely to benefit. Future studies should consider more pretesting of message content but should bear in mind that the effects may vary depending on the duration of exposure. When designing such studies, it would be prudent to consider the potential impact of intensive sampling on participant fatigue; daily monitoring may be a reasonable compromise that allows for monitoring of changes over time while reducing participant burden. Subsequent research exploring the impact of on-pack efficacy content should seek to determine the minimum level or timeframe of exposure to efficacy content and consider using HWLs with a novel format to reduce possible attentional effects of prior exposure to warning HWLs.

## Supplementary Material

A Contributorship Form detailing each author’s specific involvement with this content, as well as any supplementary data, are available online at https://academic.oup.com/ntr.

ntac229_suppl_Supplementary_Material

ntac229_suppl_Supplementary_Taxonomy-form

## Data Availability

Raw data files are available from the University of Tasmania’s data portal (https://dx.doi.org/10.25959/ypkd-pz89).
