# Supplementary material for: A Pilot Randomised Trial Investigating the Effects of Including Efficacy Messaging on Tobacco Warning Labels
Source: Nicotine Tob Res. 2022 Oct 4;25(4):773–80. doi: 10.1093/ntr/ntac229 (PMC10032187; doi:10.1093/ntr/ntac229)
Supplement: ntac229_suppl_Supplementary_Material [file ntac229_suppl_supplementary_material.pdf]

Supplementary Materials

Cognition	Items
**Self-efficacy** (1-9, totally agree – totally disagree)	I could easily cut down on the number of cigarettes I smoke.[1]
	I am confident that I will not smoke if I don't want to.[2]
	Not smoking is under my control.[2]
**Risk Perception** (1-7, from not at all – extremely)	How worried are you about your health?[3]
	Did the warning on your pack make you feel anxious? [3]
	How worried are you about the possible effects of smoking?[3]
**Intention to Quit** (1-10)	How likely do you think it is that you will actually quit smoking?[4] (likely – not likely)
	I intend not to smoke in the future[2] (totally agree – totally disagree)

Table 1. Measurement of cognitions.

1. Armitage CJ, Harris PR, Hepton G, et al. Self-affirmation increases acceptance of health-risk information among UK adult smokers with low socioeconomic status. Psychology of Addictive Behaviors. 2008 Mar;22(1):88. https://doi.org/10.1037/0893-164X.22.1.88

2. Orbell S, Lidierth P, Henderson CJ, et al. Social–cognitive beliefs, alcohol, and tobacco use: A prospective community study of change following a ban on smoking in public places. Health Psychology. 2009 Nov;28(6):753. https://doi.org/10.1037/a0016943
3. Klein WM, Harris PR, Ferrer RA, et al. Feelings of vulnerability in response to threatening messages: Effects of self-affirmation. Journal of Experimental Social Psychology. 2011 Nov 1;47(6):1237-42. https://doi.org/10.1016/j.jesp.2011.05.005
4. Sherman DA, Nelson LD, Steele CM. Do messages about health risks threaten the self? Increasing the acceptance of threatening health messages via self-affirmation. Personality and Social Psychology Bulletin. 2000 Nov;26(9):1046-58. https://doi.org/10.1177/01461672002611003
